# Nanoparticle-Encapsulated Chlorhexidine against Oral Bacterial Biofilms

**DOI:** 10.1371/journal.pone.0103234

**Published:** 2014-08-29

**Authors:** Chaminda Jayampath Seneviratne, Ken Cham-Fai Leung, Chi-Hin Wong, Siu-Fung Lee, Xuan Li, Ping Chung Leung, Clara Bik San Lau, Elaine Wat, Lijian Jin

**Affiliations:** 1 Faculty of Dentistry, The University of Hong Kong, Hong Kong SAR, China; 2 Faculty of Dentistry, National University of Singapore, Singapore, Singapore; 3 Department of Chemistry, Institute of Creativity, and Partner State Key Laboratory of Environmental & Biological Analysis, The Hong Kong Baptist University, Hong Kong SAR, China; 4 Department of Chemistry, The Chinese University of Hong Kong, Hong Kong SAR, China; 5 Institute of Chinese Medicine and Partner State Key Laboratory of Phytochemistry & Plant Resources in West China, The Chinese University of Hong Kong, Hong Kong SAR, China; LSU Health Sciences Center School of Dentistry, United States of America

## Abstract

**Background:**

Chlorhexidine (CHX) is a widely used antimicrobial agent in dentistry. Herein, we report the synthesis of a novel mesoporous silica nanoparticle-encapsulated pure CHX (Nano-CHX), and its mechanical profile and antimicrobial properties against oral biofilms.

**Methodology/Principal Findings:**

The release of CHX from the Nano-CHX was characterized by UV/visible absorption spectroscopy. The antimicrobial properties of Nano-CHX were evaluated in both planktonic and biofilm modes of representative oral pathogenic bacteria. The Nano-CHX demonstrated potent antibacterial effects on planktonic bacteria and mono-species biofilms at the concentrations of 50–200 µg/mL against *Streptococcus mutans*, *Streptococcus sobrinus*, *Fusobacterium nucleatum*, *Aggregatibacter actinomycetemcomitans* and *Enterococccus faecalis*. Moreover, Nano-CHX effectively suppressed multi-species biofilms such as *S. mutans, F. nucleatum*, *A. actinomycetemcomitans* and *Porphyromonas gingivalis* up to 72 h.

**Conclusions/Significance:**

This pioneering study demonstrates the potent antibacterial effects of the Nano-CHX on oral biofilms, and it may be developed as a novel and promising anti-biofilm agent for clinical use.

## Introduction

Dental plaque biofilm is the aetiological agent for common oral diseases such as dental caries, periodontal disease and emerging peri-implant infections [1], [2]. The pathogenic microorganisms in the plaque biofilm critically contribute to the aforementioned oral diseases.

Chlorhexidine (CHX) is a widely used antimicrobial agent in various formulations in dentistry [3], [4]. Recently, nano-encapsulation has emerged as a novel approach to delivering biologically potent compounds more effectively to specific-targets, while maintaining their original capacity in the development of new health products and drugs [5], [6]. It has been shown that nano-encapsulated drugs can enhance the overall biological effectiveness through fast penetration and bioavailability while reducing potential cytotoxicity, drug dosage and the production costs, with reference to the controls [7].

Herein, we report a novel synthesis of mesoporous silica nanoparticle encapsulated with pure (non-salt form) CHX, namely Nano-CHX; and present its morphological profile and mechanical properties. Its antimicrobial properties were comprehensively characterized by using planktonic bacteria, mono-species and mixed-species models of oral biofilms.

## Materials and Methods

### Synthesis of the Nano-CHX

The mesoporous silica nanoparticles were used and prepared according to the previously validated protocol, with an average particle diameter of around 140 nm and pore size of approximately 2.5 nm, the Brunauer, Emmett and Teller (BET) surface area of ∼1,000 m^2^/g, and pore volume ∼1.0 cm^3^/g [8]. In brief, 50 mg of pure CHX (non-salt form, Sigma-Aldrich) was dissolved in 5 mL of ethanol. Fifty milligram of mesoporous silica nanoparticles were added into 4 mL of the CHX solution and then incubated for 24 h at room temperature. This process allowed the CHX molecules to swell and assemble into the inner pores of the nanoparticles. The mixture was then centrifuged and the Nano-CHX particles were collected by membrane filtration (0.2 micron, Macherey-Nagel, Germany).

### Morphology, thermostability and release profile

The morphology of the Nano-CHX particles was analyzed by using transmission electron microscopy (TEM, Tecnai G2 20 S-TWIN). Prior to the analysis, an ethanol solution of Nano-CHX particles was drop-casted, followed by solvent evaporation at room temperature on a carbon-coated copper grid. The thermostability of the nanomaterials was analyzed by the Thermogravimetric Analyzer (Perkin Elmer TGA-6) [9]. This method determined the change of a sample weight with slowly increasing temperature, and weight loss was usually observed with a specific range of temperature. Weight percentages of CHX loaded to the blank nanoparticles were then determined by observing the weight loss at a range of 100–900°C. For detecting the release profile, the Nano-CHX (30 mg) was dispersed in 10 mL of deionized water (Barnstead RO Pure System) at 37°C for 72 h. The release profile of CHX from Nano-CHX in water over time was observed at the local maximum absorption of CHX (254 nm) by UV/visible absorption spectroscopy (Cary UV-100).

### Anti-biofilm properties in mono-species and mixed-species oral biofilms

Oral pathogenic bacteria, *viz. Streptococcus mutans* (ATCC 35668), *Streptococcus sabrinus* (ATCC 33478), *Fusobacterium nucleatum* (ATCC 25586), *Aggregatibacter actinomycetemcomitans* (ATCC 43718), *Enterococccus faecalis* (ATCC 29212) and *P. gingivalis* (ATCC 33277) were obtained from the archival collection at the Oral Biosciences, Faculty of Dentistry, The University of Hong Kong, and used for the experiments. These species were inoculated on horse blood agar plates and incubated in an anaerobic chamber with 5% CO_2_, 10% H_2_ and 85% of N_2_ at 37°C for two days. The bacterial cultures were harvested and suspended in phosphate-buffered saline (PBS) for subsequent microbiological experiments.

#### Planktonic mode

Broth microdilution assay was performed to determine the minimum inhibitory concentration (MIC) and minimum bactericidal concentration (MBC), according to the standard NCCLS criteria with slight modifications as previously described [2]. *S. mutans* and *E. faecalis* were used for this assay. Briefly, bacteria cultures were harvested and suspended with Brain Heart Infusion Broth (BHI) at a concentration of 10^6^ cells/mL using a calibrated spectrophotometer. Bacterial cultures were incubated with serially double diluted Nano-CHX particles for 48 h at 37°C under anaerobic conditions. The lowest concentration without visual bacterial growth was recorded as the MIC. Afterward, 20 µL of bacterial suspensions was inoculated in horse blood agar and kept for 48 h for further observation. The lowest drug concentration that yielded no bacterial growth was documented as MBC.

#### Mono-species biofilms

Mono-species biofilms of oral pathogens were formed according to the previous protocol [10], [11]. *S. mutans*, *S. sabrinus*, *F. nucleatum*, *A. actinomycetemcomitans* and *E. faecalis* were resuspended in BHI at a concentration of 10^8^ cells/mL to develop a biofilm, respectively. 100 µL of standard cell suspension was pipetted to polystyrene 96-well plates and incubated under anaerobic conditions for 24 h. The biofilms were washed with PBS and treated with a diluted series of Nano-CHX for 24 h before taking MIC readings. In parallel, planktonic bacterial cell suspension at 10^8^ cells/mL was used to obtain the MIC readings.

#### Mixed-species biofilms

Three selected combinations of mixed-species were made, *viz.* i) *S. mutans* with *F. nucleatum* and *P. gingivalis*; ii) *S. sobrinus* with *F. nucleatum* and *P. gingivalis*; and iii) *S. mutans* with *F. nucleatum*, *A. actinomycetemcomitans* and *P. gingivalis*. These mixed-species biofilms were formed by mixing equal amount of bacterial suspensions as an initial inoculum and they were grown under anaerobic conditions for 24, 48 and 72 h as described above. Following each time point, the biofilms were treated with Nano-CHX for 24 h using blank nanoparticles as the negative controls. The MIC of Nano-CHX against the mixed-species biofilms was determined by treating the biofilms with Nano-CHX as described. During this experiment, it was observed that Nano-CHX was unable to eradicate the mature biofilm at 72 h. Therefore, another assay was performed to examine the preventive potential of Nano-CHX against the mixed-species biofilms. In this assay, Nano-CHX was introduced to the wells of the plate together with mixed-species bacterial inoculum. Biofilm growth was monitored for 24, 48 and 72 h.

### Microscopic observations of the efficacy of Nano-CHX against oral biofilms

In a separate set of experiments, the mixed-species biofilms of *S. sobrinus*, *F. nucleatum* and *P. gingivalis* were developed in 1×1 cm sterilized coupons made of polystyrene material (IWAKI, Tokyo, Japan). The biofilms were formed on coupons in a 12-well plate (IWAKI, Tokyo, Japan) under anaerobic conditions as described above. After 24 h, the biofilms were treated with Nano-CHX particles for 24 h. Blank nanoparticles were used as the controls. These specimens were subjected to Scanning Electron Microscopy and Confocal Scanning Laser Microscopy as described previously [11]. Experiments were repeated in three separate occasions.

#### Scanning Electron Microscopy (SEM) analysis

Each coupon with the mixed-species biofilms was post-fixed in a 5% gluteraldehyde and 3% formaldehyde solution (5% v/v 0.1 M phosphate buffer, pH 7.4) for 4 h, air dried, and then placed in 1% osmium tetroxide for 1 h. Coupons were subsequently washed in distilled water, dehydrated in a series of ethanol washes (70% for 10 mins, 95% for 10 mins and 100% for 20 mins), and air dried in a desiccator. Afterward, the specimens were mounted on aluminum stabs with copper tape, coated with gold in a low-pressure atmosphere with an ion sputter coater (JEOL JFC1 100: JEOL, Tokyo, Japan). The topographic features of each biofilm were visualized with a SEM (Hitachi S-3400N SEM at EM Unit, HKU) in high vacuum mode at 10 kV.

#### Confocal Scanning Laser Microscopy (CSLM)

Molecular Probes' Live/Dead BacLight Viability kit comprising SYTO-9 and propidium iodine (PI) (Molecular Probes, Eugene, OR) was used for the CSLM analysis of the mixed-species biofilms and selected mono-species biofilms of *S. mutants*, *S. sobrinus* and *E. faecalis*. The Control and Nano-CHX treated biofilms were washed once with 1.5 mL of PBS and stained with Live/Dead BacLight Viability kit using previously established protocol by our group [11]. In brief, coupons were incubated with SYTO-9 and PI for 30 min in the dark at 37°C. Subsequently, coupons were mounted on microscope slides and the images of stained biofilms were captured using a CSLM system (FLUOVIEW FV 1000, Olympus, Tokyo, Japan). All tests were repeated three times.

### Statistical analysis

The statistical significance of MIC and MBC values of Nano-CHX against both planktonic and biofilm modes as well as different species were analyzed by Student's t-test or ANOVA as appropriate by using IBM SPSS Statistics for Windows, Version 21.0, IBM Corp. Armonk, NY, USA.

## Results

### Characterization of the Nano-CHX particles

**Figure 1:**
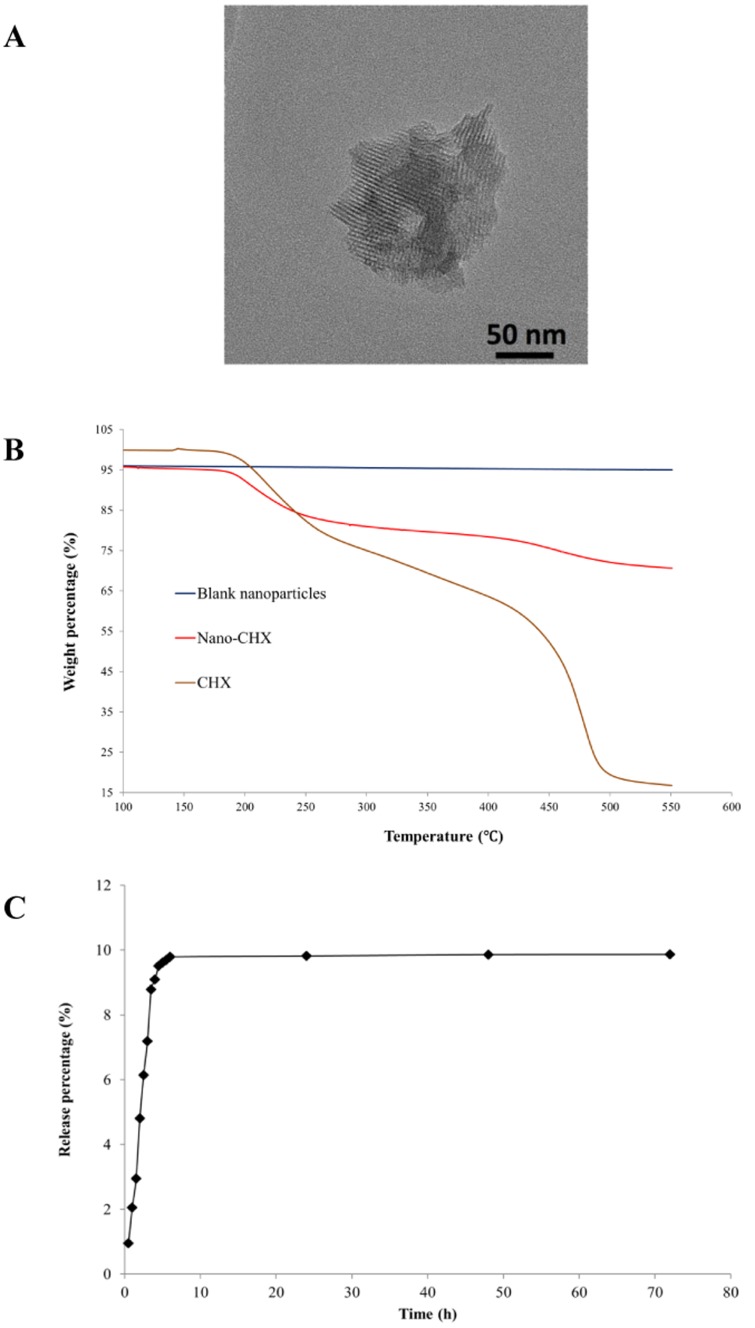
The characteristics of the Nano-CHX particles. A transmission electron microscopic image of single nanoparticle (A). The thermogravimetric analysis on the weight losses of i) CHX between 150–500°C (80.6%); ii) blank nanoparticles between 150–500°C (1.3%); and iii) Nano-CHX between 150–500°C (23.1%) (B). The release profile of CHX (%) from the Nano-CHX assessed by UV/visible absorption spectroscopy at 254 nm (C).

The transmission electron microscopic image showed that a single blank nanoparticle exhibited ordered and structured inner porous channels with around 2.5 nm diameter (Figure 1A). As shown in Figure 1B, the thermogravimetric analyses revealed that a significant weight loss (80.6%) of CHX was observed from 150 to 500°C, and the weight loss of blank nanoparticles was 1.3% between 150–500°C. As the weight loss of nanoparticles Nano-CHX was 23.1% between 150–500°C, the CHX loading efficiency was calculated to be 21.8% after a background subtraction with the blank nanoparticles. This finding demonstrated that a significant amount of CHX was capable to be loaded onto the nanoparticles by swelling and assembling processes. Furthermore, the release experiment showed a quick and marked release of CHX from Nano-CHX that reached a maximal saturation of around 10% at 6 h, and this saturated level of released CHX persisted till the end of the observation period of time at 72 h (Figure 1C).

### Antimicrobial activity against planktonic and biofilm modes of oral pathogens

The MICs of Nano-CHX against *S. mutans* and *E. faecalis* were 19.5 µg/mL and 156 µg/mL, respectively; while the respective MBC values were 312.5 µg/mL and 1250 µg/mL. Notably, the blank nanoparticles did not demonstrate any antimicrobial activity (5000 µg/mL was ineffective). Hence, the antimicrobial activity of Nano-CHX was due to the action of CHX on the microorganisms, instead of the residual effect of nanoparticles *per se*. Comparative analysis of the mono-species biofilm and planktonic modes (10^8^ cells/mL inoculum) showed a promising antibacterial activity of Nano-CHX treatment for 24 h (Table 1). Interestingly, the Nano-CHX treatment (100 or 200 µg/mL) for 24 h was highly effective against the mono-species biofilms, including *S. mutans*, *S. sobrinus*, *F. nucleaturm*, *A. actinomycetemcomitans* and *E. faecalis*. The CLSM images of Nano-CHX-treated (24 h) mono-species biofilms of *S. mutants*, *S. sobrinus* and *E. faecalis* are presented in Figure S1.

**Table 1:** The minimal inhibitory concentration (MIC, µg/mL) of Nano-CHX against the planktonic mode and mono-species biofilms of pathogenic oral bacteria at 24 h.

Bacterial species	Planktonic mode	Mono-species biofilms
*S. mutans*	50	100**
*S. sobrinus*	100	200*
*F. nucleatum*	50	100**
*A. actinomycetemcomitans*	50	100**
*E. faecalis*	100	200*

Significant difference from the planktonic mode,

*p<0.05,

**p<0.01.

**Figure 2:**
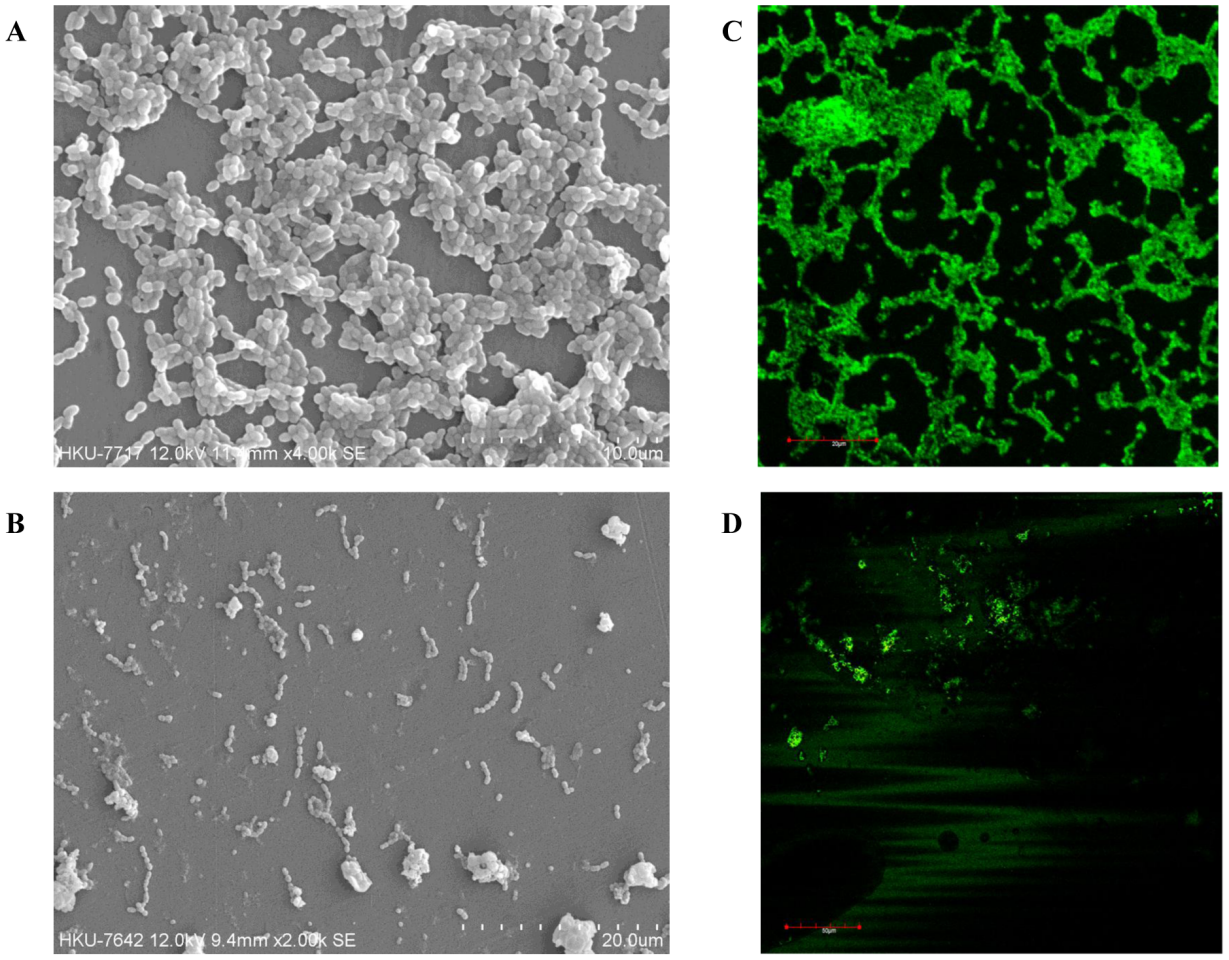
The antibacterial effects of Nano-CHX treatment for 24 h on the mixed-species biofilms of *S. sobrinus*, *F. nucleatum* and *P. gingivalis*. Representative scanning electron microscopy images: B vs. A (blank nanoparticles); and confocal laser scanning microscopy images: D vs. C (blank nanoparticles).

Furthermore, in the mixed-species biofilm assay it was observed that the Nano-CHX was unable to eradicate the 72 h mixed-species biofilm. Hence, we proceed to observe the preventive action of Nano-CHX against mixed-species biofilms. The MIC data showed a potent antibacterial activity of Nano-CHX against all three groups of mixed-species biofilms at 24, 48 and 72 h (Table 2). Microscopic observations using SEM and CLSM further confirmed the above observation on the mixed-species biofilms of *S. sobrinus*, *F. nucleatum* and *P. gingivalis*. As shown in the SEM and CLSM images, only a few scattered bacterial cells (Figure 2B), or a few viable cells existed (Figure 2D) existed, with reference to the control treated with the blank nanoparticles (Figures 2A and 2C).

**Table 2:** The minimal inhibitory concentration (MIC, µg/mL) of the preventive effect of Nano-CHX on the mixed-species oral biofilms in a time-dependent assay from 24 to 72 h.

Mixed-species biofilms	24 h	48 h	72 h
*S. mutans*, *F. nucleatum* & *P. gingivalis*	12.5	50	50
*S. sobrinus*, *F. nucleatum* & *P. gingivalis*	25	50	100
*S. mutans*, *F. nucleatum*, *A. actinomycetemcomitans* & *P. gingivalis*	25	50	50

## Discussion

CHX is well known to be effective against both Gram-positive and Gram-negative bacteria [4], [12]. It is also less likely to develop antimicrobial resistance as compared to antibiotics, and hence serves as a good candidate for further developing new antimicrobial products [13]. In the present study, we attempted to develop novel Nano-CHX particles and examine their antibacterial effects on both planktonic and biofilm modes of representative oral pathogenic bacteria, such as *S. mutans*, *S. sobrinus*, *E. faecalis*, *F. nucleatum*, *A. actinomycetemcomitans* and *P. gingivalis*. These bacteria are significantly involved in common oral diseases, such as dental caries, pulpal infections and periodontal disease.

In the present study, the mesoporous silica nanoparticles with inner pore channels of approximately 2.5 nm were used [9], [14], [15] for loading of pure CHX. By swelling the blank mesoporous nanoparticles in an ethanolic solution of pure CHX, a significant amount of over 20 weight percent of CHX was encapsulated in the nanoparticles. Theoretically, the salt forms of CHX such as CHX gluconate, chloride and acetate may not achieve the loading efficacy as high as the pure CHX (21.8%), mainly due to their relatively large molecular size. Moreover, the assembly process of CHX toward the nanoparticles proceeded smoothly. Notably, the Nano-CHX particles were capable of being dried and well re-dispersed in water without jeopardizing the structure of these particles. The silica material employed in this study has recently been shown to be relatively non-cytotoxic [7], [16]–[18]. This material has also been clinically approved to be used as an implant material [19]. Therefore, we assume that the novel Nano-CHX could have a promising clinical usage, although more *in vitro* and *in vivo* studies are highly warranted for affirmative evidence.

One of the major concerns with antimicrobial agents in clinical setting is the growth mode of microbial biofilms [20]. Numerous studies have shown that the biofilm mode of microbial growth can be highly resistant to antimicrobial agents with reference to their planktonic counterparts [21], [22]. Pathogenic plaque biofilms consisting of mixed pathogens are the etiological agent for major oral diseases like dental caries and periodontal diseases [1], [2]. Over the years, CHX containing oral healthcare products such as mouth rinses have been frequently used in the clinical practice, due to its board-spectrum antimicrobial properties [3], [23], [24]. On the other hand, studies have also shown the limitation of CHX formulations owing to its inability to effectively penetrate the mature dental plaque biofilm and low substantivity [25], [26]. A recent study demonstrates that CHX is more effective against *de novo* biofilms and its effects may reduce with time [27]. Hence, the substantivity of CHX only persisted for 6–10 h after mouth rinse, and the salivary and plaque bacteria gradually obtained a higher viability with time [28], [29]. The foregoing fact highlights the need to develop novel delivery vehicles for slow-release and relatively high penetration of CHX with increased efficiency and effectiveness against oral biofilm for better clinical usage. Therefore, great attention has been increasingly paid to develop improved application form of CHX by incorporating nanotechnology. However, there are only a very few studies assessing the efficacy and effectiveness of Nano-CHX against oral pathogens. For instance, a recent study has shown that Nano-CHX particles could be effective against *S. mutans* biofilms *in vitro* [30].

Interestingly, the novel Nano-CHX particles developed in the present study demonstrate promising antimicrobial effects against both planktonic and biofilm bacteria. Hence, Nano-CHX is effective against major oral pathogens such as *S. mutans*, *S. sobrinus*, *F. nucleatum*, *A. actinomycetemcomitans* and *E. faecalis* at 50–100 µg/mL for their planktonic modes and at 100–200 µg/mL for their mono-species biofilms, respectively. Moreover, our study further shows that the Nano-CHX at 12.5–100 µg/mL can inhibit the development of multi-species oral biofilm up to 72 h, and this finding has been supported by SEM and CLSM observations on the mixed-species biofilms of *S. sobrinus*, *F. nucleatum* and *P. gingivalis*. Technically, the finding from the present release experiment also shows a maximal saturated release of CHX from Nano-CHX at 6 h without consumption, and the anticipated further release of CHX could persist against microorganisms. Further study is required to confirm this point. It is worthy to note that in the present study *P. gingivalis* was selected as a pathogen in the three groups of multi-species oral biofilms for the experiments. It is worth noting that *P. gingivalis* has been implicated as a keystone bacteria, through facilitating a critical change in the composition of normal oral microbiota and modulating immuno-inflammatory response, thereby contributing to the development of periodontal disease and tissue destruction [31]–[33]. Based on the positive results observed, it could be assumed that the Nano-CHX may be able to penetrate effectively the oral biofilms and control the bacteria. Further intensive study is certainly required to determine the exact effects of Nano-CHX on different forms of multi-species biofilms and the underlying mechanisms of antimicrobial actions, with reference to CHX alone. The present ‘proof of concept’ study on the Nano-CHX may lay a foundation for further *in vitro* and *in vivo* studies on this promising novel antimicrobial agent.

In summary, the mesoporous silica nanoparticle has been effectively loaded with CHX, and its effective release from the generated Nano-CHX has been confirmed. The MIC and MBC of the Nano-CHX have been evaluated on the planktonic mode of pathogenic oral bacteria, mono-species bacterial biofilms and multi-species oral biofilms, respectively. Its anti-biofilm effects have been characterized by SEM and CLSM. In conclusion, this pioneering study demonstrates the potent antibacterial effects of the Nano-CHX on oral biofilms, and it may be developed as a novel and promising anti-biofilm agent for clinical use.

## Supporting Information

Figure S1 **The antibacterial effects of Nano-CHX treatment for 24 h on the selected mono-species biofilms.** Representative confocal laser scanning microscopy images of blank nanoparticles- and Nano-CHX-treated mono-species biofilms of *Streptococcus mutants* (A vs. D), *Streptococcus sobrinus* (B vs. E) and *Enterococcus faecalis* (C vs. F), respectively.
(TIF)
