# Supplementary figures and images for: Nanoparticle-Encapsulated Chlorhexidine against Oral Bacterial Biofilms
Source: PLoS One. 2014 Aug 29;9(8):e103234. doi: 10.1371/journal.pone.0103234 (PMC4149348; doi:10.1371/journal.pone.0103234)

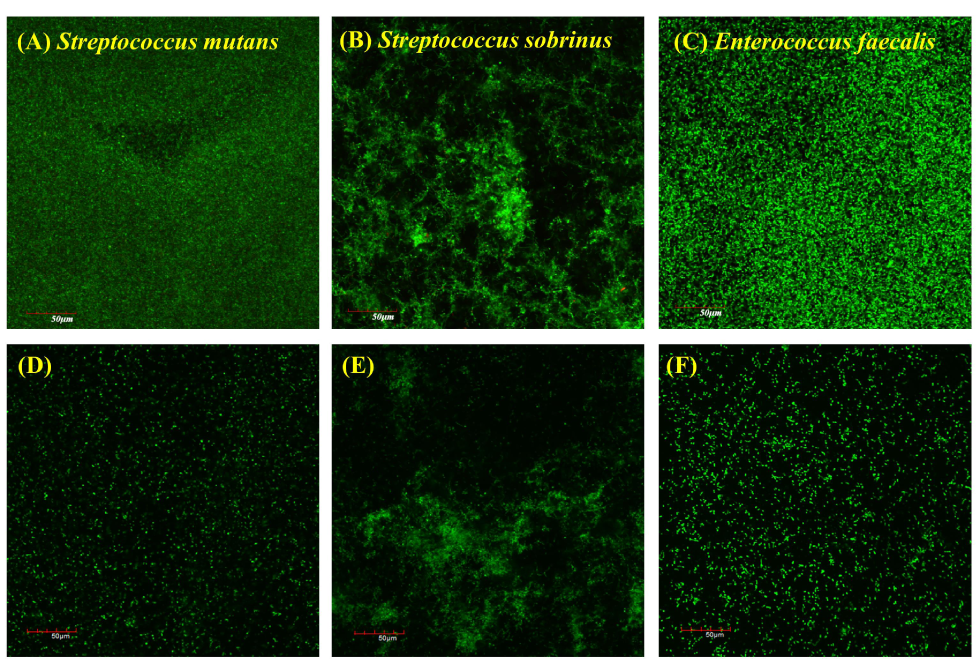

Supplement: Figure S1 — The antibacterial effects of Nano-CHX treatment for 24 h on the selected mono-species biofilms. Representative confocal laser scanning microscopy images of blank nanoparticles- and Nano-CHX-treated mono-species biofilms of Streptococcus mutants (A vs. D), Streptococcus sobrinus (B vs. E) and Enterococcus faecalis (C vs. F), respectively. (TIF) [file pone.0103234.s001.tif]
